# The Voltage-Dependent Anion Channels (VDAC) of *Mycobacterium avium* phagosome are associated with bacterial survival and lipid export in macrophages

**DOI:** 10.1038/s41598-017-06700-3

**Published:** 2017-08-01

**Authors:** Lia Danelishvili, Jessica J. J. Chinison, Tuan Pham, Rashmi Gupta, Luiz E. Bermudez

**Affiliations:** 1 Department of Biomedical Sciences, College of Veterinary Medicine, Corvallis, OR USA; 2 Department of Microbiology, College of Science, Corvallis, OR USA; 3 Department of Biochemistry and Biophysics, College of Science, Oregon State University, Corvallis, Oregon, 97331 USA; 4 College of Medicine, University of Central Florida, Orlando, Florida 32827 USA

## Abstract

*Mycobacterium avium* subsp. *hominissuis* is associated with infection of immunocompromised individuals as well as patients with chronic lung disease. *M. avium* infects macrophages and actively interfere with the host killing machinery such as apoptosis and autophagy. Bacteria alter the normal endosomal trafficking, prevent the maturation of phagosomes and modify many signaling pathways inside of the macrophage by secreting effector molecules into the cytoplasm. To investigate whether *M. avium* needs to attach to the internal surface of the vacuole membrane before releasing efferent molecules, vacuole membrane proteins were purified and binding to the surface molecules present in intracellular bacteria was evaluated. The voltage-dependent anion channels (VDAC) were identified as components of *M. avium* vacuoles in macrophages. *M. avium* mmpL4 proteins were found to bind to VDAC-1 protein. The inactivation of VDAC-1 function either by pharmacological means or siRNA lead to significant decrease of *M. avium* survival. Although, we could not establish a role of VDAC channels in the transport of known secreted *M. avium* proteins, we demonstrated that the porin channels are associated with the export of bacterial cell wall lipids outside of vacuole. Suppression of the host phagosomal transport systems and the pathogen transporter may serve as therapeutic targets for infectious diseases.

## Introduction

*Mycobacterium avium* subsp. *hominissuis* (*M. avium*) is the most common pathogen among non-tuberculosis mycobacteria, and of great public health relevance as one of the leading cause of bacterial infection in patients with HIV/AIDS as well as in individuals with chronic lung conditions^1, 2^. The opportunistic pathogen has the ability to invade and proliferate within a variety of mammalian cells including mucosal epithelium cells and macrophages. Following uptake, the pathogen is contained within a cytoplasmic vacuole, and intracellular survival is dependent on a number of bacterial virulence determinants used to remodel the vacuolar compartment and to resist the host antimicrobial mechanisms^3–6^. *M. avium* can prevent the recruitment of proton-ATPase to the vacuole and, therefore, inhibits the acidification of the phagosome^7^. The pathogen arrests the maturation of phagosomes in the early endosome phase^8^ by interfering with trafficking process^5^, and grow in non-acidified compartments^9^. *M. avium* actively survives and resists the most effective cellular killing mechanisms by molecules of reactive oxygen intermediates (ROIs) and nitric oxide (NO)^10–12^. Another characteristic of *M*. *avium* is the ability to use apoptosis as a trigger to escape from phagocytes and infect surrounding cells^13, 14^. The interaction between virulent mycobacteria and host antimicrobial mechanisms is assumed to be an active process controlled only by a viable bacilli, since none of above effects occur following phagocytosis of dead mycobacterium or after inhibition of bacterial protein synthesis^15, 16^.

The specialized protein secretion systems are one of the main virulence determinants of pathogenic bacteria that efficiently deliver bacterial secreted effectors directly to the cytosol across eukaryotic membranes, either plasma or vacuolar. Many pathogens coordinately deliver/inject virulence factors via Type III, IV and/or VI secretion machineries to the extracellular (tissues or bloodstream) or intracellular (host cells) environment. Mycobacteria lack all of above virulence-associated secretion machineries, and in addition they are encapsulated in an unique lipid-rich mycolate layer. An increasing body of literature indicate that mycobacterium protein export is facilitated in part by the Type VII secretion system (T7SS), which plays a central role in mycobacterial pathogenesis^17, 18^. Pathogenic mycobacteria species encode up to five copies (ESX1–5) of T7SS, and disruptions of the T7SS systems or their substrates have been shown to diminish bacterial intracellular fitness or decrease in virulence^3, 4, 19^. The best-characterized ESX-1 locus of RD1 is involved in the secretion of ESAT-6 and CFP-10 of *Mycobacterium tuberculosis* and *Mycobacterium marinum* ^20, 21^ influencing the host cell signaling and cytokine secretion^22^ and apparently required for the escape of *M. tuberculosis* from the phagolysosome into the cytosol^23^. *M. avium*, that lacks the ESX-1 region, has been demonstrated to use the ESX-5 system for virulence. The ESX-5 locus exports several extracellular proline-glutamic acid proteins, the PPE and PE virulence factors^4, 24^, found within the mycobacterial cell envelope^25^ and characterized by the antigenic variation and consequent immune evasion^26, 27^. Studies have demonstrated that many PE/PPE proteins found in *M. avium* are secreted and the disruption of PE/PPE family genes is linked to bacterial attenuation^3, 4^.

Despite the significant progress made in the past decade, it is still unknown how mycobacteria translocate virulence effectors through the membrane-bound phagosome and deliver effector molecules into the cytosol of the host cell. Since intracellular mycobacterium is found juxtaposed to the phagosome membrane, the goal of this study was to identify possible phagosomal proteins that are employed by *M. avium* to export virulence factors into the cytosol of host cells.

## Results

### VDAC porins are associated with *M. avium* phagosomes

**Figure 1:**
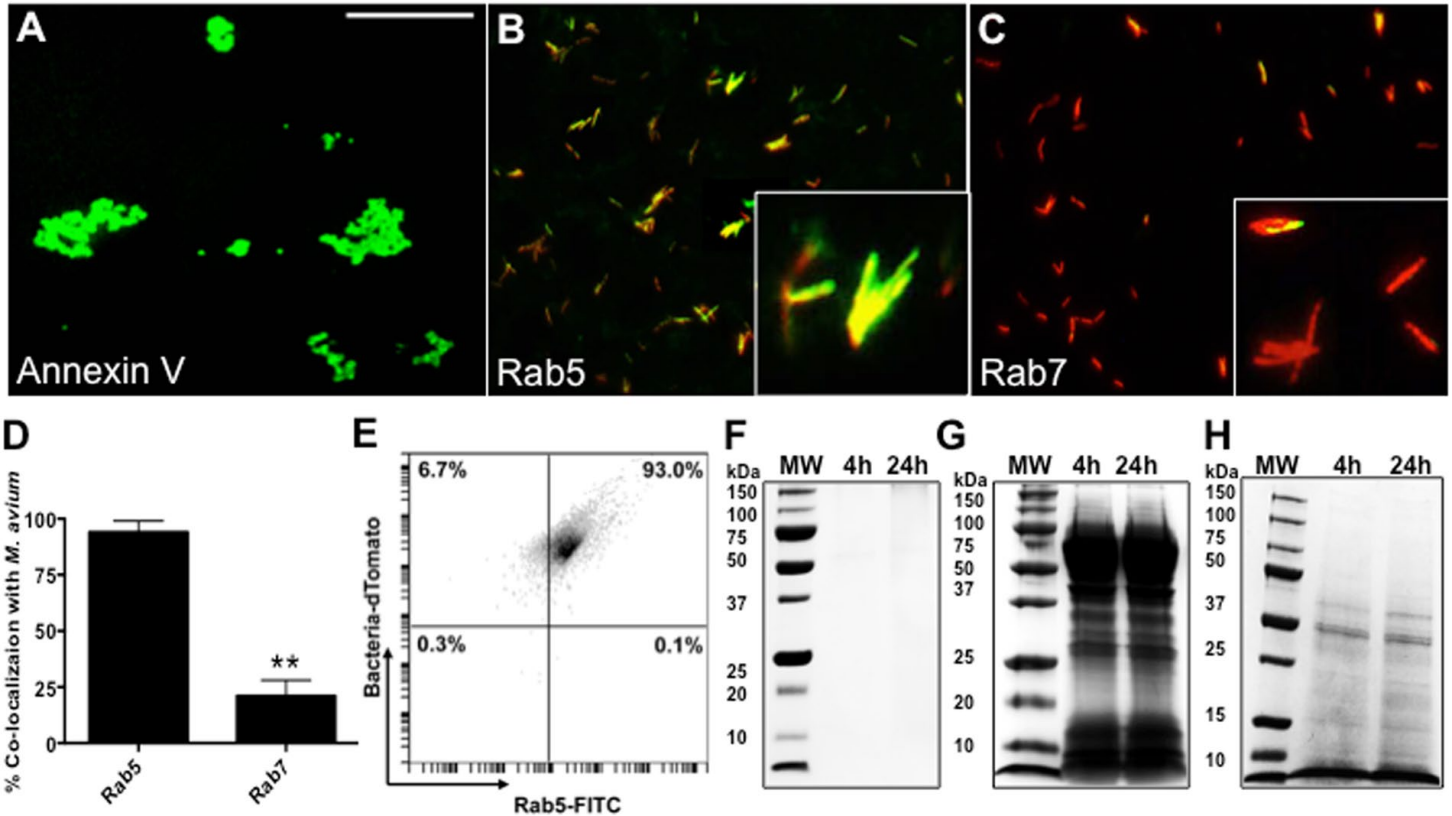
Magnetically labeled *M. avium* and isolation of phagosomes. The intact phagosomes of biotin labeled tomato red clone of *M. avium* were separated from the total THP-1 cells lysate using the streptavidin-coated MACS microbeads as described in Materials and Methods. The labeled phagosomes with the Alexa Fluor 488-conjugated Annexin B **(A)** Rab5 **(B)** and Rab7 **(C)** were visualized for purity under the fluorescent microscopy. Scale bar 5μm. *M. avium*-containing phagosmes were stained with antibodies against Rab5 or Rab7 for 2 h at a dilution of 1:250 in PBS containing 3% BSA. After washing, phagosomes were probed with FITC-conjugated secondary antibody for 1 h and then processed for fluorescence microscopy. (**D**) The percentage of co-localized tomato red-labeled *M. avium* and FITC-labeled Rab5 and Rab7 phagosomal markers was determined by evaluating three hundred bacterial cells and express as the mean ± SD for three separate experiments. Significant differences were observed between Rab5 and Rab7 in their co-localization with the *M. avium* phagosome. **p < 0.001. The dtTomato *M. avium*-containing phagosomes stained for Rab5 were analyzed by flow cytometry as well **(E)**. To verify the purity of intracellular *M. avium* sample and rule out the contaminant host proteins, bacteria isolated from human macrophages at 4 h and 24 h post-infection were incubated with the extraction buffer for 2 h with gentle agitation. The resulting supernatants **(F)** and the host cell total proteins of infected THP-1 cells (used for isolation of the intracellular *M. avium*) were visualized on a protein gel with the Coomassie staining **(G)**. The magnetically purified *M. avium* phagosomes were lysed in 20 mM HEPES supplemented with the 1% Tergitol and protease inhibitor cocktail and visualized on the SDS-PAGE (**H**).

*M. avium* phagosomes were purified using biotin labeling and magnetic purification technique, previously described for mycobacteral phagosomes^28^. After magnetic separation, the intact phagosomes isolated from infected THP-1 cells were stained with Alexa Fluor 488 conjugated Annexin V (Fig. 1A), Rab5 (Fig. 1B) and Rab7 (Fig. 1C) markers, and examined under fluorescence microscopy. To visually determine Rab5 and Rab7 labeled phagosomes, we evaluated three hundred bacterial cells expressing the tomato red protein and the percentage of positive phagosomes was calculated. In agreement with previous studies^29^, the most of *M. avium*-containing phagosomes were positive for Rab5, whereas the co-localization of bacteria with Rab7 was significantly less seen (Fig. 1D). Using a BD accuri C6 flow cytometer, the phagosomes were further assessed for flow cytometry and the Rab5 co-localization with labeled bacteria was observed in 93% of isolated phagosomes (Fig. 1E).

In order to identify the phagosomal proteins interacting with the surface of *M. avium* in the host environment, the adherence of vacuolar proteins to the intracellular *M. avium* was assayed. To insure that the isolated intracellular bacterial sample did not contain contaminant non-phagosomal proteins, the intracellular *M. avium* isolated at 4 h and 24 h time-points were resuspended in the extraction buffer (20 mM Octyl β-D-glucopyranoside and 25 mM EDTA; Sigma) and incubated for 2 h on a rotator at 4 °C. Resulting supernatants (Fig. 1F) as well as the host cell total protein fraction (Fig. 1G) of THP-1 cells were separated by SDS-PAGE and visualized by Coomassie staining. The proteins of phagosomal lysates are presented in the Fig. 1H. The crude phagosomal extract was incubated with *M. avium*, as described in the material and methods, and the unbound proteins were removed by washing bacterial cells three times in PBS. Elution of bound proteins from the bacterial surface with the extraction buffer yielded 33 proteins (Table 1). The mass spectrometric analysis identified many previously described phagosomal proteins such as ATP synthase, prelamin, prohibitin, anexin A5 and vimentin^30^. Unexpectedly, there were many mitochondrial proteins identified raising a possibility for the localization of mitochondrial proteins onto the phagosomal membrane. Interestingly, all three members of the eukaryotic mitochondrial porin ion channels or Voltage-Dependent Anion Channels (VDAC-1, VDAC-2 and VDAC-3) were found to be associated with *M. avium* surface. In order to eliminate the possibility that washing the bacterial surface with light detergent of 20 mM Octyl β-D-glucopyranoside and 25 mM EDTA, previously described in similar studies^6, 31^, did not result in *M. avium* cell lysis, the obtained mass spectrometric (MS) data were analyzed against mycobacterial database. Only seven proteins listed in the Table 2 including two mmpL4 (mycobacterial membrane protein large 4) lipoproteins, known to be localized on the surface of *M. avium*, were identified in the sample. To extract the bacterial surface proteins, more vigorous techniques such as partial trypsinization or selective labelling of surface proteins and affinity purification have to be applied for mycobacteria^32^. In addition, we performed the control experiment where the pellet of 7H9 Middlebrook broth grown *M. avium* was washed twice with HBSS and then incubated with the extraction buffer for 2 h. The mass spectrometric analysis of the resulting sample confirms that the incubation with the extraction buffer does not lead in bacterial cell lysis or in striping the bacterial surface (data not shown). This observation raised a possibility that identified *M. avium* proteins listed in the Table 2 most likely formed complexes with some of phagosomal proteins. This phenomenon was further confirmed in this study.

**Table 1:** Phagosomal proteins bound to *M. avium* surface identified by the mass spectrometric sequencing.

#	Identified Human Proteins	Accession Number	MW kDa	Peptides	
				4 h	24 h
1	Cluster of Vimentin	VIME_HUMAN [3]	54	26	36
2	Prelamin-A/C	LMNA_HUMAN (+1)	74	24	26
3	ATP synthase subunit beta, mitochondrial	ATPB_HUMAN	57	19	20
4	ATP synthase subunit alpha, mitochondrial	ATPA_HUMAN	60	11	12
5	Prohibitin	PHB_HUMAN	30	12	10
6	Cluster of ADP/ATP translocase 2	ADT2_HUMAN	33	7	12
7	Heterogeneous nuclear ribonucleoprotein A3	ROA3_HUMAN	40	11	8
8	Cluster of Histone H2A (Fragment)	H0YFX9_HUMAN [12]	10	10	10
9	U5 small nuclear ribonucleoprotein 200 kDa helicase	U520_HUMAN	245	11	7
10	Annexin A5	ANXA5_HUMAN (+1)	36	8	9
11	ATP-dependent RNA helicase A	DHX9_HUMAN	141	6	8
12	Splicing factor 3B subunit 3	SF3B3_HUMAN	136	8	6
13	Voltage-dependent anion-selective channel protein 1	VDAC1_HUMAN	31	11	6
14	60 S acidic ribosomal protein P2	RLA2_HUMAN	12	7	8
15	Cluster of Histone H2B	B4DR52_HUMAN [11]	18	6	6
16	Heterogeneous nuclear ribonucleoprotein M	HNRPM_HUMAN	78	7	3
17	Histone H4	H4_HUMAN	11	7	6
18	Prohibitin-2	J3KPX7_HUMAN (+1)	33	7	4
19	60 S ribosomal protein L4	RL4_HUMAN	48	5	7
20	Heterogeneous nuclear ribonucleoproteins A2/B1	ROA2_HUMAN	37	5	6
21	Splicing factor 3B subunit 1	SF3B1_HUMAN	146	6	4
22	Cluster of Heterogeneous nuclear ribonucleoprotein L	HNRPL_HUMAN [2]	64	6	5
23	Pre-mRNA-processing-splicing factor 8	PRP8_HUMAN	274	7	3
24	Heterogeneous nuclear ribonucleoprotein A1	F8VZ49_HUMAN (+2)	26	5	4
25	Voltage-dependent anion-selective channel protein 2	B4DKM5_HUMAN (+1)	27	4	7
26	116 kDa U5 small nuclear ribonucleoprotein	K7EJ81_HUMAN (+1)	108	5	4
27	60 S ribosomal protein L9 (Fragment)	D6RAN4_HUMAN (+2)	21	6	3
28	Cluster of 60 S acidic ribosomal protein P0 (Fragment)	F8VU65_HUMAN [3]	27	4	6
29	rRNA 2’-O-methyltransferase fibrillarin	FBRL_HUMAN	34	5	2
30	60 S ribosomal protein L10a	RL10A_HUMAN	25	5	5
31	Voltage-dependent anion-selective channel protein 3	F5H740_HUMAN (+1)	31	4	4
32	Cluster of Heterogeneous nuclear ribonucleoprotein H2	HNRH2_HUMAN [2]	49	3	4
33	Polypyrimidine tract-binding protein 1	PTBP1_HUMAN	57	5	3

**Table 2:** *M. avium* proteins identified in phagosomal protein fraction bound to bacterial surface.

#	Identified *M. avium* Proteins	Accession	MW kDa	Peptides	
				4 h	24 h
1	MmpL4 protein, MAV_4696	A0QLN5	107	4	2
2	2-C-methyl-D-erythritol 4-phosphate cytidylyltransferase, ispD	A0QAB3	23	2	0
3	Putative transport protein MmpL4, MAV_0084	A0Q8Z4	106	2	0
4	Transcriptional regulator, TetR family protein, MAV_2167	A0QEN8	20	0	2
5	2-hydroxy-6-ketonona-2,4-dienedioic acid hydrolase, MAV_2517	A0QFM11	32	0	2
6	Dehydrogenase, MAV_3890	A0QJG41	56	0	2
7	Acyl-CoA synthase, MAV_0030	A0Q8U2	54	2	0

### Inhibition of VDAC results in reduction of bacterial viability in THP-1 cells

**Figure 2:**
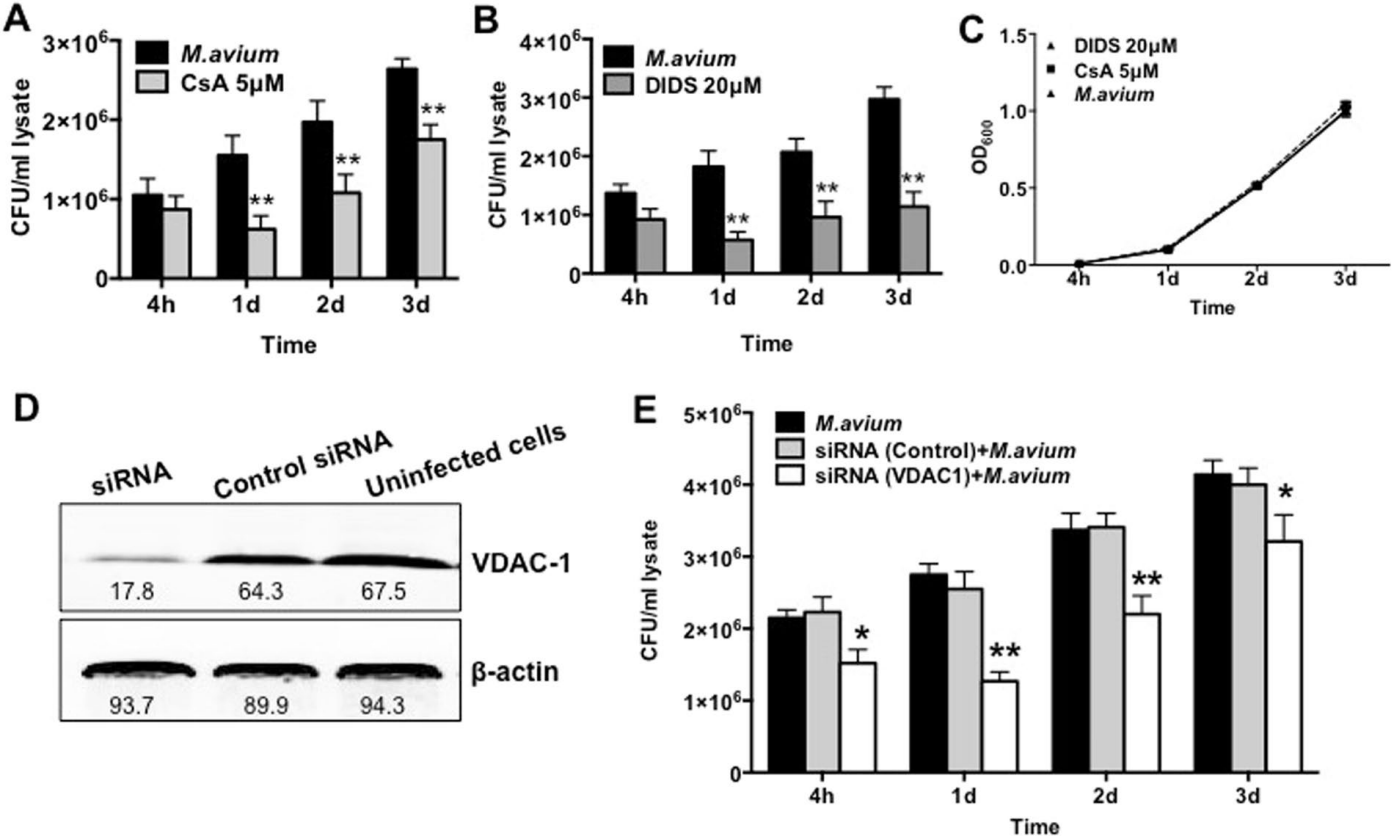
Suppression of *M. avium* growth in macrophages during VDAC inhibition. (**A)** THP-1 cells were pretreated with 5 μM CsA and then infected with *M. avium* up to 3 day; cultures were harvested at indicated time points and bacterial number were determent by CFU counts. Results represent mean ± standard error of three independent experiments. **p < 0.01, the significance of differences between CsA treated and control groups at the corresponding time points. (**B**) Macrophages were pretreated and cultured in DIDS or control medium. Bacterial number was determined at 4 h, and 1, 2, and 3 days post-infection by plating cell lysates on 7H10 agar plates. The difference in DIDS-treated THP-1 cultures compared with untreated cells was statistically significant at day 1, 2 and 3 (P < 0.01, t test). Data are representative of three experiments. **(C)** *In vitro* growth of *M. avium* in aerated 7H9 medium containing DVAC inhibitors at concentrations used for tissue culture infection studies. **(D)** THP-1 cells (approximately 10^5^/ml) were seeded and differentiated into macrophages with PMA in 6-well plate. After 24 h, cells were replenished with new medium and allowed to rest additional 48 h. Macrophage monolayers were washed with siRNA transfection medium and replaced with either the VDAC-1 siRNA transfection reagent or the scrabbled sequences of negative control siRNA 24 hours prior *M. avium* infection. Briefly, cells were lysed in CelLytic™ M lysis buffer supplemented with protease inhibitor cocktail (Sigma) and pre-cleared samples were separated on 12% Tris–HCl gels. Membranes were blocked with 3% BSA for 1 h and incubated with VDAC-1 primary antibody at a 1:250 dilution for 2 h. After, membrane was probed with the corresponding IRDye® secondary antibody (Li-Cor Biosciences, Inc) at a dilution of 1:5,000 for 30 min. THP-1 cells transfected with VDAC-1 siRNA for 72 h demonstrates efficient and specific silencing of VDAC-1 quantified via semi-quantitative western blot on the Odyssey Imager (Li-Cor). The photon emission means were recorded for each band to quantify the signal intensity. Beta-actin was used as a loading control. **(E)** Intracellular CFU decrease at several times of post-infection of transfected THP-1 cells with VDAC-1 siRNA or siRNA control and infected with *M. avium*. Data are means ± SD of three independent experiments. **p < 0.01 and *p < 0.05, the significance of differences between VDAC-1 knock down and siRNA control or *M. avium* infection groups.

To investigate the relationship between VDAC and *M. avium* virulence, we inhibited channel proteins by pretreating THP-1 cells with 5 μM Cyclosporine A (CsA), a potent blocker of VDAC complex. Macrophages were treated with CsA 4 hours prior bacterial infection to avoid long incubation with these inhibitors and to prevent adverse effects and triggering functional imbalance in the host cells. While *M. avium* was able to enter and infect the host cells at the same rate (treated as well as untreated control), the chemical impairment of VDAC function had significant effect on bacterial growth at 1, 2 and 3 days post-infection when compared with untreated group as determined by the number of bacterial CFU (Fig. 2A).

Previous studies have suggested that the process of oligomerization of VDAC proteins led to the formation of large pores allowing for release of the proapoptotic factors such as cytochrome C out of mitochondria^33^. To investigate the possibility that VDAC oligomerization in *M. avium* phagosome membrane took place and was involved in the translocation of larger molecules into the cytoplasm, we examined the bacterial survival in macrophages that were pretreated with the 4,4′-Diisothiocyano-2,2′-disulfonic acid stilbene (DIDS), a blocker of VDAC oligomerization, 4 hours prior *M. avium* infection. The CFUs were recorded over 3 days following infection. The results from the inhibition of VDAC by DIDS displayed similar ability of *M. avium* to invade THP-1 cells as untreated control group. However, *M. avium* had impaired capability to grow and survive in monolayers that were treated with DIDS, and significant decrease in growth was observed at day 1, 2 and 3 time points when compared with the control group (Fig. 2B).

To rule out toxic effects of CsA or DIDS on *M. avium* growth *in vitro*, approximately 1 × 10^6^ bacteria were cultured in 7H9 broth with or without VDAC inhibitors and OD readings were monitored over 3 days. Results indicate that CsA at 5 μM and DIDS at 20 μM had no inhibitory or killing effect on bacterial growth *in vitro* (Fig. 2C).

### Inhibition of the VDAC-1 gene expression is associated with suppression of *M. avium* growth in THP-1 cells

The mass spectrometric analysis of *M. avium* phagosomes identified VDAC-1 channels as the most abundant channel present in the sample. Using the small interfering RNA (siRNA), we were able to suppress the expression of VDAC-1. Western blot result shows that VDAC-1 expression was significantly reduced compared to control siRNA and untreated cells (Fig. 2D). To verify the differences in *M. avium* survival in macrophages with functional VDAC-1 and in cells with impaired gene expression, intracellular bacteria were cultured from phagocytic cells and CFUs were recorded at several time points following infection. As observed in the Fig. 2E, while reduced *M. avium* growth was observed at 4 h in VDAC-1-inactivated macrophages, differences were even more significant at 1 and 2 days post-infection compared with THP-1 cells transfected with the scrambled siRNA control. *M. avium* was able to recover on day 2 and 3, however, bacterial growth in VDAC-1-silenced monolayers continued to lag behind when compared with scrambled siRNA controls at the same time points.

### *M. avium* proteins interacting with VDAC-1

**Figure 3:**
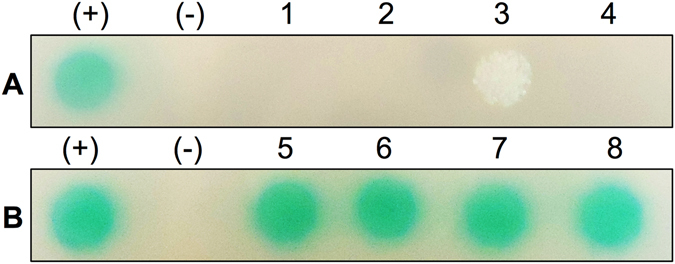
*In vitro* protein-protein interaction between VDAC-1 and *M. avium* effectors. The open reading frame of VDAC-1 cDNA encoding a 283 amino acid protein was amplified from the human sequence-verified Clone ID: 6023095 (Dharmacon), which was isolated from the NIH_MGC library. The yeast two-hybrid interaction of VDAC-1 with the bacterial target proteins MAV_1177 (1), MAV_2921 (2), MAV_2941 (3) and CipA (4) showed a negative or false positive interaction. The screening of mmpL4 lipoproteins MAV_0084 (5) and MAV_4996 (6) as well as ATP synthase alpha (7) and beta (8) subunits established positive interaction with VDAC-1. The known interaction between pGBKT7-53 and pGADT7-T served as a positive control (+), whereas pGBKT7-lam and pGADT7-T were used as a control for a negative interaction (−).

We hypothesized that VDAC channels may play a role in the export of bacterial proteins into the cytosol of host phagocytic cells. To examine this hypothesis, we studied interactions between VDAC-1 and selected *M. avium* secreted effectors (MAV_1177, MAV_2921, MAV_2941 and CipA) using the yeast two-hybrid system. Previous studies identified some of these proteins to be secreted into the cytoplasm of host cells^3, 5^ while CipA is secreted upon contact with cell surface^34^. None of these effectors showed to have positive interaction with the channel, as the resulting zygotes of both the bait and pray constructs did not grow in the absence of Ade, His, Leu, and Ttp and presence of 125 ng/ml Aureobasidin and X-a-Gal. An exception was MAV_2921; however, the yeast MAV_2921 clone did not turn blue in the presence of X-a-Gal meaning that the transcription of the α-galactosidase reporter gene *MEL-1* did not take place, giving the false interaction result with VDAC-1 (Fig. 3A).

We then performed the pull-down assay to expand our search in finding *M. avium* proteins that might interact with VDAC-1. Only two *M. avium* proteins, ATP synthase subunit alpha and beta were found to bind VDAC-1 (Table 3). The further investigation through the yeast two-hybrid system, shown in the Fig. 3B, has proved the specificity of the interaction of both subunits of the ATP synthase.

**Table 3:** *M. avium* proteins bound to VDAC-1 identified by pull-down assay.

#	Identified Proteins	Accession	MW kDa	Peptides
1	ATP synthase subunit alpha, MAV_1525	ABK67333	59	2
2	ATP synthase subunit beta, MAV_1527	ABK66662	53	6

The mmpL4 proteins were identified in the *M. avium* surface-bound phagosome fraction using mass spectrometric analysis. Due to the fact that mmpL proteins participate in export of mycobacterium cell wall components to the bacterial surface and genetic interferences affect pathogen fitness *in vitro* and *in vivo* ^35^, we examined the yeast two-hybrid interaction between mmpL4 lipoproteins (MAV_0084 and MAV_4996) and VDAC-1, finding it to be positive (Fig. 3B).

### Immunostaining reveals co-localization of VDAC-1 with mmpL4

**Figure 4:**
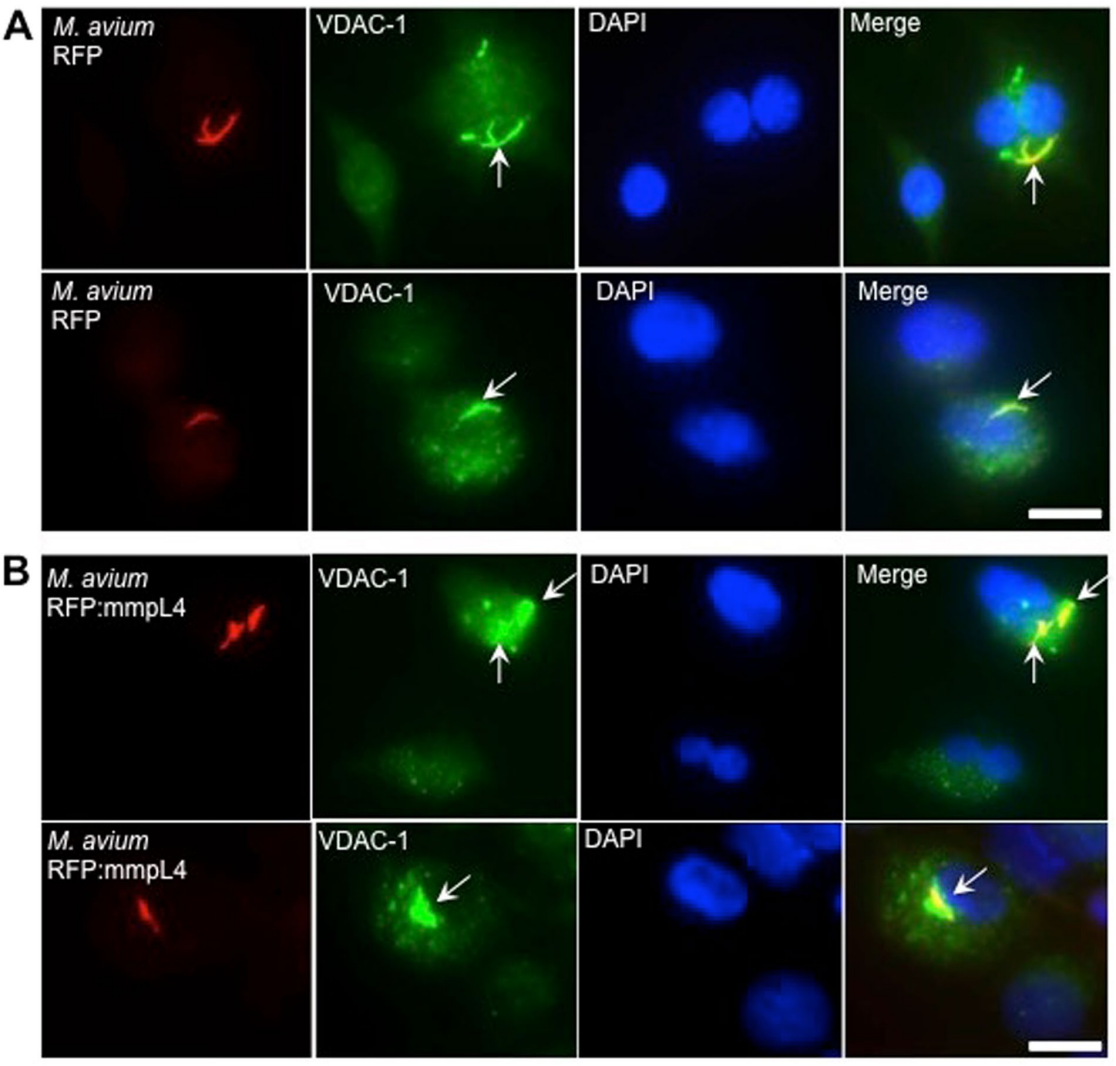
The co-localization of VDAC-1 on phagosomes of *M. avium* expressing mmpL4 protein. Representative images of constitutively expressing RFP **(A)** and RFP:mmpL4 **(B)** proteins in *M. avium* show subcellular co-localization of VDAC-1 on bacterial vacuoles. The arrows highlight specific regions of interest visualizing the overlapping yellow pixel clusters (co-localization). Images contain uninfected control cells as well. All images were obtained using 100x oil objective of a fluorescent microscope (Leica). Nuclei were stained with 4,6-diamidino-2-phenylindole (DAPI). Two images are included for each experimental group. Bar = 10 μm.

We also performed immunofluorescence staining of VDAC-1 in THP-1 cells that were infected with either a *M. avium* clone containing the Red Fluorescent Protein (RFP) or a clone overexpressing mmpL4 (MAV_4696) protein in fusion with RFP. While the granular fluorescence of VDAC-1 protein was dispersed in the cytosol of uninfected cells (Fig. 4A and B), *M. avium* infected cells showed punctate staining on bacterial vacuoles (Fig. 4A). VDAC-1 staining in infected THP-1 cells revels that this channel protein is always localized with bacterial-containing phagosomes. The fact that the phagosome membrane is originated from the host cell plasma membrane during the infection process and VDAC-1 is one of the components of the plasma membrane^36, 37^, may explain the observation. In addition, the VDAC-1 was stained with a greater intensity on *M. avium* vacuoles overexpressing the mmpL4 protein (Fig. 4B) than in control macrophages (Fig. 4A), suggesting the host protein co-localization with this bacterial surface protein.

### The role of VDAC in *M. avium* cell wall lipid release in macrophages

**Figure 5:**
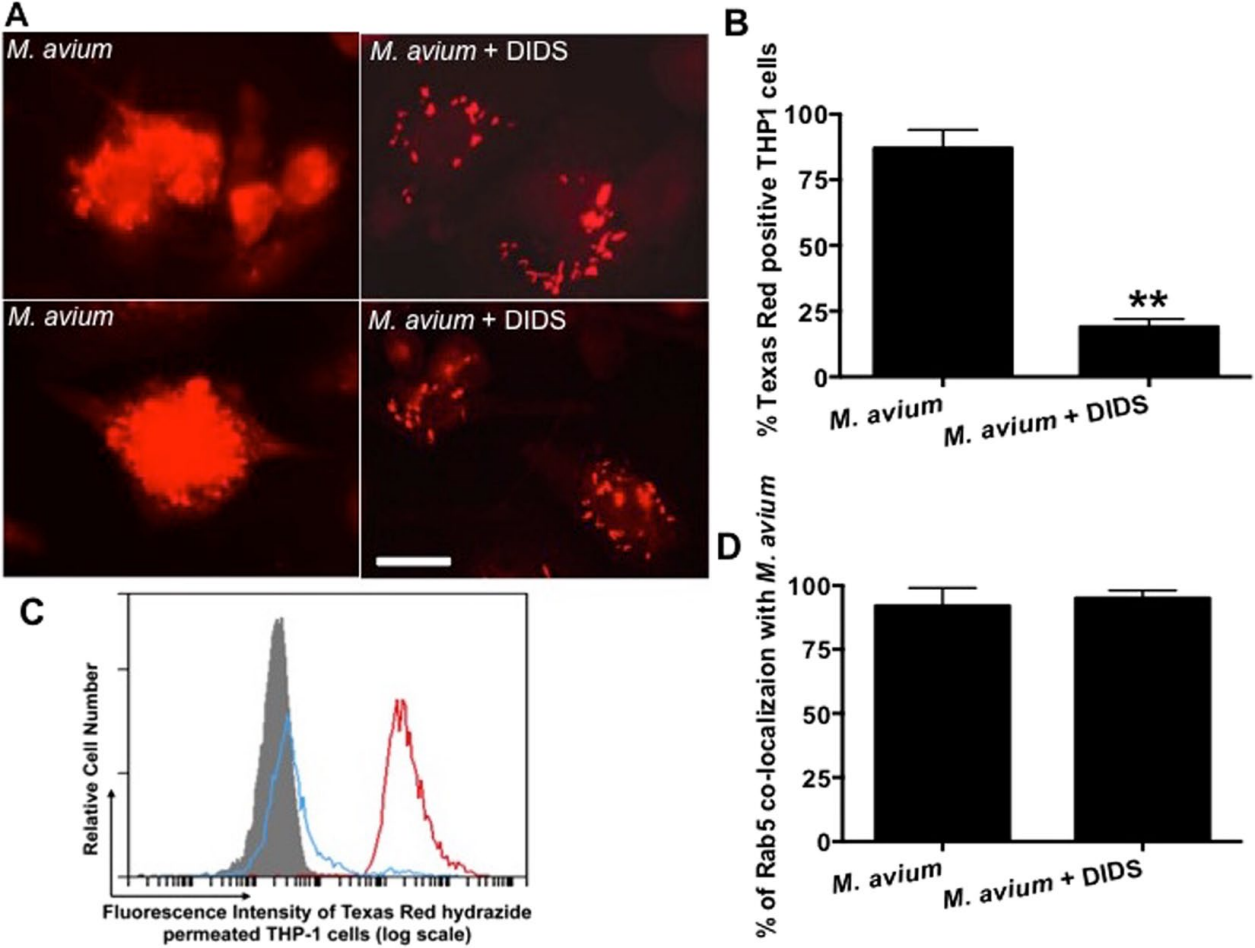
*M. avium* cell wall lipid release inside of macrophages. **(A)** THP-1 cells with or without DIDS treatment were infected with Texas Red hydrazyde-labeled *M. avium* with MOI of 25:1 for 24 h and analyzed by fluorescent microscopy. While significant release of fluorescent label from bacterial phagosomes are observed in wells without DIDS treatment, the export of bacterial cell wall components into the cytosol of macrophages are substantially reduced as observed on micrographs obtained from infected THP-1 cells during VDAC inhibition. Two images are included for each experimental group. Scale bar 10μm. **(B)** The percentage of the host macrophages permeated the red fluorescence released from the Texas Red hydrazyde-labeled *M. avium*. Results represent means ± standard error of three independent experiments. *, *p* < 0.001, the significance of differences between *M. avium* infected THP1 cells with and without DIDS treatment. **(C)** *M. avium* infected THP-1 macrophages with DIDS (blue trace) or without DIDS (red trace) treatment were analyze by flow cytometry to discern lipid transport as described in the materials and methods. The host cells without infection are shaded grey. **(D)** To visualize and demonstrate the colocalization of Rab5 with the Texas Red hydrazide stained *M. avium* directly in THP-1 infected cells without DIDS treatment was technically impossible, due to the massive release of lipids within the host cells. Thus, the percentage of *M. avium* co-localization with Rab5 phagosomal marker was determined by evaluating three hundred *M. avium*-containing phagosmes, which were isolated from THP-1 cells with and without DIDS treatment at 24 h post-infection as described in materials and methods. Results represent means ± standard error of two independent experiments.

Mycobacterial mmpL proteins have been well documented to be involved in the biosynthesis and export of cell wall lipid constituents, and play a role in mycobacterium pathogenesis^38^. In addition, recent studies on VDAC have generated strong evidence on its association/interaction with host lipids^39, 40^. The ability of VDAC to influence the cholesterol distribution of mitochondrial membrane has been also recently demonstrated^41^, and cholesterol and ergosterol have been found to form complex with purified VDAC protein^42^. It also has been established that the oligomerization of VDAC can be significantly influenced by lipids^40^. In attempts to investigate the possible relation between VDAC, mmpL4 proteins and *M. avium* surface-associated lipid export into macrophages, we pretreated THP-1 cells with DIDS for 4 hours and then infected cells with Texas red hydrazide-labeled *M. avium*. The DIDS was kept up to 24 h in the culture medium and lipid release from bacterial surface was analyzed by fluorescent microscopy. THP-1 cells without DIDS treatment served as a control. As previously identified by Beatty *et al*.^15^, the extensive release of the Texas red label from mycobacterial surface was observed at 24 h post-infection of THP-1 (Fig. 5A). In contrast, macrophages treated with DIDS had the red fluorescent label markedly contained within *M. avium* phagosomes, suggesting the involvement of VDAC in bacterial cell wall component translocation. Evaluation of two hundred *M. avium*-infected THP-1 cells without DIDS treatment confirmed the observation that majority (87%) of the host macrophages permeated the red fluorescence that was released from the Texas Red-labeled bacteria. Conversely, only 19% of the DIDS treated macrophages had a positive staining (Fig. 5B). Results were further confirmed using the flow cytometry (Fig. 5C). To insure that the fluorescent labeling of host cells was not the result of *M. avium* presence in the cytosol, the percentage of Rab5 positive phagosomes were calculated in THP-1 cells with and without DIDS treatment and the co-localization rate of Rab5 in both groups were observed to be similar (Fig. 5D).

## Discussion

*M. avium*, like many other pathogenic mycobacteria, is highly adapted for survival within phagocytic cells. In the vacuolar compartments, bacteria are isolated from a rich source of nutrients existing in the cytoplasm. The phagosome membrane separates the intravacuolar bacteria from the cytoplasm and therefore is placed between released virulence factors and targets in the host cell cytoplasm. Many studies have demonstrated that *M. avium* as well as *Mycobacterium tuberculosis* secrete virulence factors inside the vacuole environment^5, 15, 20, 43^. Differently from gram-negative bacteria, such as *Salmonella*, mycobacteria do not have classical type III or type IV secretion systems to inject effector molecules across membranes. Both *M. avium* and *M. tuberculosis* are equipped with the type VII secretion system which is a major export mechanism for proteins belonging to the ESX regions^3, 17^. *M. tuberculosis* also uses a pili-like structure to secrete two proteins involved in apoptosis inhibition of macrophages^44^. If other proteins employ the above mentioned secretion apparatus as well, it is currently unknown. Therefore, in the absence of an “injectosome”, such as the types III and IV secretion systems, *M. avium* depends of an alternative approach to deliver the virulence-associated molecules to the cytoplasm of macrophages.

In this study, we investigated the vacuole membrane transport mechanisms that are used by the pathogen to be able to translocate secreted molecules across the vacuole membrane. We hypothesized that bacterial effectors would recognize a counterpart on the vacuole membrane facilitating the transport of exported products. In fact, our screen identified a large number of host proteins on the phagosome membrane that are recognized by bacterial surface structures. The majority of the models of phagocytosis describe the plasma membrane as the main source of the phagosomal membrane formation. Several proteins identified in our study have been previously demonstrated to be part of the plasma and phagosomal membranes^30^. The mass spectrometric sequencing, however, also identified several mitochondrial proteins on *M. avium* phagosomes. The fact is that pathogens such as *Salmonella*, mycobacteria and *Leishmania* avoid the degradation into phagolysosomes by inhibiting the fusion of the phagosomes with the endocytic organelles^45^. The vacuolar compartments of pathogens such as *Brucella* ^46^ and *Chlamydia* ^47^ receive lipids from the trans-Golgi network and end up having the endoplasmic reticulum or Golgi features on the phagosomes. Previous proteomics studies have also reported the presence of mitochondrial proteins in phagosomal membrane^48, 49^. Although it is still difficult to define the role of many organelle proteins in the vacuole membrane, evidence suggests that the plasma membrane is probably not the only source of phagosomal membrane and some select class of mitochondrial proteins may also contribute to formation of phagosomes. Among mitochondrial proteins previously shown to be part of the phagosome^30^, VDAC proteins are strong candidates for translocation of bacterial-derived molecules. VDAC are voltage-channels that initially were described as mitochondria-associated porins responsible for ATP/ADP exchange, and involved in the flux of metabolites across the mitochondrial outer membrane^50, 51^. More recently, these channels have been identified on the plasma membrane^36, 37^, and in phagosome membranes of latex beads, *M. bovis* BCG and Brucella vacuoles^30, 52, 53^. Studies have shown the ability of VDAC to bind to and transport cholesterol, and influence its distribution between the inner and outer mitochondrial membranes^41, 54^. The VDAC was found to allow the translocation of DNA sequences across a planar membrane^55^. In addition, the transport of large molecule such as the cytochrome C across the mitochondrial membrane^56^ was accomplished following fusion of several VDAC molecules to form a large pore known as an oligomerization process^57^.

In order to examine if VDAC had a role in the transport of *M. avium* secreted proteins, first we selected known effector proteins to be exported in the cytosol of macrophages and investigated protein-protein interaction using the yeast two-hybrid system. We also performed the pull-down assay, however, only two *M. avium* proteins of alpha and beta subunits of ATP synthase (ATPases) were found to bind VDAC-1. These interactions were further confirmed with the yeast two-hybrid system and the binding of the host VDAC-1 molecule to bacterial ATPases were found to be positive. Previous studies have described the association of ATPases with the surface of intracellular *M. avium* ^32^ and at the mycobacterial surface during biofilm formation (Rose at). *M. tuberculosis* ATPases function in the cell envelope^58^ providing energy for substrate transport^59^ and driving type VII protein export across the cytoplasmic membrane^60^. On the other hand, the interaction between host VDAC and ATPases, and regulation of ATP trafficking in and out of the mitochondria has been well documented^61^. The above facts strongly support our finding that bacterial ATPases can be associated with VDAC and possibly are involved in this channel gating. This hypothesis, however, requires further confirmation in the experimental systems.

We were unsuccessful to demonstrate that bacterial secreted proteins employ the VDAC system as a mechanism of transport. During our investigation, however, it became clear that the function and oligomerization of VDAC are crucial for *M. avium* growth within phagosomes of the host macrophage. We were able to demonstrate that VDAC-1 protein co-localizes and interacts with *M. avium* mmpL4 proteins. MmpL family proteins are unique to the mycobacterial core genome, and a growing body of literature indicates that the primary role of most mmpL proteins are dedicated to transport of mycobacterial lipids for incorporation into the cell wall^35^. Inactivation of many of these genes leads to failure to export the mycolic acid-containing lipids and mycolate ester wax to the bacterial surface. The lipid export function has been described for mmpL3, mmpL7, mmpL8 and mmpL11. The recent study suggests that mmpL3 transport trehalose out of the cell wall, and its inhibition prevents the incorporation of *de novo* synthesized mycolic acids into the cell wall^62^. In fact, using the β-lactamase reporter transposon, Dr. Braunstein’s group has mapped the exported protein domains of MmpL4^63^. The location as well as the identity of mmpL4 transporter substrates has not been fully elucidated, however, the functional studies suggest that mmpL4 is involved in the biosynthesis of cell surface polyketides and the glycopeptidolipids^64^ and most likely is juxtaposed to the cell wall as the majority of the mmpL family proteins.

Beatty and colleagues^15^ demonstrated that mycobacterial lipids are released from the bacterial phagosome and accumulate in late endosomal/lysosomal compartments of macrophages. Due to the fact that bacterial lipids were also found in extracellular milieu and subsequently internalized by uninfected neighboring macrophages, the authors raised the possibility that mycobacterial exported lipids most likely have an immunomodulatory effect contributing to the control of surrounding uninfected cells. This hypothesis was later confirmed by O’Neil and colleagues^65^. On the other hand, it has been shown that the presence of specific host lipids can change VDAC conformational equilibrium and regulate the voltage gating of the channel^66^. VDAC is also capable to bind and transport the host lipids^41, 54^. In this study, we examined whether blocking the VDAC oligolimerization process had any inhibitory effect on *M. avium* lipid export. Indeed, we observed the significant decrease in bacterial lipid export in host macrophages during DIDS treatment when compared with the untreated control. Currently, it is unknown whether VDACs are the only channel-forming proteins associated with the translocation of mycobacterial lipids. Previous studies using the morphological and biochemical analysis of phagosomes of isolated latex beads identified the VDAC as one of the component of the phagosome membrane^30^. The presence of VDAC on phagosomes of Bacille Calmette-Guerin (BCG)^53^ and Brucella-infected macrophages^52^ raises the possibility that the transport mechanism may be common among some pathogens. All these observations, including our study, suggest that the VDAC proteins previously identified in other cellular compartments are representative of more than a simple contamination and the VDAC molecules are genuine constituents of phagosomes.

Mycobacteria inside the macrophage vacuole appear to use host cell transport system to translocate virulence factors into the cytoplasm. Our finding is in agreement with the observation by de Chastellier and colleagues^67^ who discovered that the contact between bacteria and phagosome membrane is required for *M. avium* survival in macrophages. Our data suggests that at least in some cases, the export of bacterial constituents begins with the recognition of a transport system in the vacuole membrane by a *M. avium* mmpL4 proteins. Recent report indicated that treatment of *M. tuberculosis*-infected macrophages with cyclosporin A protects mitochondria from the mitochondrial permeability transition^68^. This process blocks the host cell necrosis induced by this pathogen and shifts to apoptotic death enhancing antimycobacterial activity of macrophages and killing of intracellular *M. tuberculosis*. While it may be the only explanation, we also want to highlight that our observation raises another possibility. In the *M. avium* model, the inhibition of apoptosis and induction of necrosis do not occur, and therefore bacterial attenuation in the macrophage is unlikely to be explained by the cell necrosis. Furthermore, the use of siRNA and the absence of observation of necrosis in monolayers exposed to the inhibitor and control monolayers, ruled out the possibility.

In the current study, we demonstrate that the VDAC transport system interacts with mmpL4 proteins on the vacuole membrane of *M. avium*, and functional channels are required for the pathogen survival in macrophages. The underlying mechanism of interaction between bacterial ATPases and VDAC molecules is still unknown, but based on the existing research literature there is a possibly that ATPases may regulate the channel function. In this work, we can conclude that *M. avium* alters the VDAC function in a pathogen-directed manner. The pathogen translocates bacterial lipids via VDAC system and inhibition of the oligomerization process of the VDAC channel contributes to the dynamic changes of mycobacterial intraphagosome and, thus, *M. avium* survival within the phagocyte. Understanding the molecular basis of phagosome channels, its regulation and activation mechanism most likely will have a crucial importance for designing new therapeutic tools against mycobacterial diseases.

## Materials and Methods

### Bacterial strain and hydrazide labeling

*Mycobacterium avium* strain 104 was originally isolated from the blood of AIDS patients with disseminated infection. Bacteria were maintained in Middlebrook 7H9 broth (BD Biosciences) supplemented with 10% (vol/vol) olei acid-albumin-dextrose-catalase (OADC; Hardy Diagonstics) at 37 °C for 6 days. The tomato red clone of *M. avium* 104 strain was created suing pJDC60 mycobacterial plasmid expressing the tdTomato gene under L5 promoter provided by Dr. Jeffrey Cirillo at Texas A&M University System Health Science Center, College Station, TX. This clone was maintained in the Middlebrook culture medium supplemented with 400 μg/ml kanamycin. Mycobacterium surface-exposed terminal oxidizable carbohydrates were labeled with hydrazide according the protocol published by Beatty *et al*.^15^. Before labeling, bacterial cells were washed twice with PBS containing 0.05% Tween 80, and resuspended in 0.1 M sodium acetate and 1 mM sodium periodate (Sigma) solution at pH 5.5. *M. avium* was gently rotated for 20-min at 4 °C and then the reaction was stopped by adding 0.1 mM glycerol. Bacterial cell suspension was washed three times with PBS supplemented with 0.05% Tween 80 followed by 2 h incubation in PBS/Tween containing 1 mM Texas Red hydrazide (Molecular Probes) at room temperature. The culture was washed twice and, prior infection, the bacterial viability was determined by colony forming units (CFU) on Middlebrook 7H10 agar.

### Cell culture maintenance and infection

The THP-1 human monocyte cell line was purchased from the American Type Culture Collection (ATCC) and maintained in Roswell Park Memorial Institute medium (RPMI; Corning) supplemented with 10% (vol/vol) fetal bovine serum (FBS; Gemini) in 75 cm^3^ flasks. Prior infection, cells were differentiated by adding 5ng/ml of phorbol 12-myristate 13-acetate (PMA, Sigma Aldrich) to culture medium and, depending on experiment performed, were seeded in range of 60–95% confluence into 6-, 24-well plates, two-chamber glass slides or T-200 tissue culture flasks. Following 24 h incubation at 37 °C in an atmosphere of 5% CO_2_, cell culture medium was replenished with fresh new medium and incubated for additional 48–72 h for cell differentiation. Macrophages were infected with mid-log phase grown *M. avium* and after two hours post-infection, wells were extensively washed with the Hank’s Balanced Salt Solution (HBSS, Life technologies), and the total number of viable bacteria in the inoculum as well as cell-associated bacteria over time were determined by CFU counts. In all experiments, except infections with the hydrazide-labeled bacteria, the multiplicity of infection (MOI) was adjusted to ~10 bacteria per macrophage.

### Magnetic isolation of intact phagosomes

The mid-log phase grown *M. avium* 104 in Middlebrook 7H9 broth were pelleted, washed twice with HBSS and passed 10 times through a 27-gauge needle to ensure a single cell suspension. *M. avium* was incubated at room temperature with 1 mg/ml EZ-Link sulfo-NHS- LC biotin (Thermo Fisher Scientific) in PBS for 30 minutes. The reaction was stopped by washing bacterial pellet with PBS supplemented with 0.1 M glycine at pH 7.2, and then the pellet was resuspended in PBS with 0.05% Tween-80 to remove unbound biotin. Biotinylated *M. avium* was incubated under gentle agitation with streptavidin-coated microbeads (Miltenyi Biotech) for 20 minutes at room temperature. Macrophages were seeded up to 95% confluence in T-200 flasks and infected with labeled *M. avium* at MOI of 10:1. After 4 h and 24 h incubation at 37 °C 5% CO_2_, macrophages were scraped and resuspended in homogenization buffer (1 M HEPES with 1 M sucrose; Life Technologies) containing protease inhibitors cocktail (Sigma). THP-1 cells were then mechanically lysed by multiple passages through a 27-gauge needle. Intact phagosomes were selected through a MiniMACS column on a magnetic selector obtained from Miltenyi Biotech and the bound phagosomes were eluted in PBS. Isolated phagosomes were incubated with Alexa Fluor 488- conjugated Annexin B (Thermo Fisher Scientific) at a dilution of 1:1,000 and visualized on a Leica DM4000B micriscope. In addition, Rab5 and Rab7 phagosome markers were immunostained using anti-Rab5 and anti-Rab7 mouse monoclonal antibodies (Santa Cruz Biotechnology) at a dilution of 1:500 followed by visualization with corresponding FITC conjugated secondary antibody (1:1,000). Three hundred bacterial cells expressing the tomato red protein were evaluated to calculate the percentage of positive phagosomes for either Rab5 or Rab7. Purified phagosomes were further processed for protein purification as follows: phagosomes were resuspended in 1% Tergitol (Sigma) in 20 mM HEPES (Sigma) supplemented with the protease inhibitor cocktail (Sigma) and lysed overnight. Twenty-four hours later, the suspension was centrifuged to remove bacteria and microbeads, and protein sample was processed for electrophoresis and Coomassie staining.

### Isolation of *M. avium* surface bound phagosomal proteins

The intracellular, non-biotin labeled, *M. avium* 104 was extracted from THP-1 cells at 4 h and 24 h time-points of infection as previously described^69^. To assess if samples had any host cell protein contaminants, isolated bacteria were washed twice in cold HBSS, and then incubated in the extraction buffer (20 mM Octyl β-D-glucopyranoside and 25 mM EDTA; Sigma) for 2 h on a rotator at 4 °C. Resulting supernatants were separated by SDS-PAGE and visualized by Coomassie staining.

Isolated phagosomal proteins were combined with the intracellular *M. avium* and incubated at 4 °C. After 24 h, bacterial pellet was centrifuged at 3,500 rpm for 20 min, washed three times with PBS and resuspended in the extraction buffer to elute any phagosomal protein that was bound to the surface of the intracellular *M. avium*. The bacteria were pelleted down and collected supernatant was processed for the buffer exchange procedure using 3 kDa filters and the 25 mM ammonium bicarbonate as the exchange buffer. Eluted phagosomal proteins were trypsin digested in solution at 37 °C for 5 h and sequenced by electrospray ionization mass spectrometry (ESI-MS/MS) at the Oregon Health Science University (OHSU) proteome facility.

### Construction of mmpL4 overexpression *M. avium* clone

To demonstrate binding of bacterial mmpL4 protein to VDAC-1, the full length MAV_4696 gene was cloned into *Hind*III site of pMV261–5HRFP^3^ as C-terminal fusions to a monomeric RFP moiety with an N-terminus 6X-His tag. Vector construction and gene cloning confirmation were performed in *E. coli*. Vectors with and without mmpL4 gene were transformed into *M. avium* and selected on Middlebrook 7H10 agar containing kanamycin 400 μg/ml. Resulting red colonies were selected for immunostaining experiments. Following infection of THP-1 cells, we analyzed bacterial and host protein co-localization with fluorescent microscopy.

### Immunofluorescent microscopy

Approximately, 1 × 10^5^ THP-1 cells were seeded in 2-chamber slides and infected with hydrazide-labeled bacteria with MOI of 25 bacteria to 1 cell. Following 4 h and 24 h infection, cells were fixed in 4% formaldehyde for 30 min. VDAC-1 antibody was purchased from the Santa Cruz Biotechnology, Inc and used at 1:100 dilution followed by visualization with corresponding FITC conjugated secondary antibody (1:1,000). Slides were mounted and observed under a Leica DM4000B fluorescent microscope (Leica).

### Effect of VDAC inhibitors, cyclosporine A and 4,4′-Diisothiocyano-2,2′-disulfonic acid stilbene, on *M. avium* growth

Two widely used inhibitors for VDAC channels: cyclosporine A (CsA; Novartis), an inhibitor of the CA^2+^- dependent VDAC pore (Lobatón *et al*., 2004; Yuqi *et al*., 2009), and 4,4′-Diisothiocyano-2,2′-disulfonic acid stilbene (DIDS), a blocker of VDAC oligomerization, were selected to impair the channel function. Prior macrophage inhibition assays, we tested effects of CsA (5 μM) and DIDS concentrations (20–200 μM), used in tissue culture studies, on *M. avium* viability. Bacteria were incubated with 5 μM CsA and 20–200μM-concentration range of DIDS and CFUs were recorded at 4 h, 1d, 2d, and 3d post-infection. Five micromole CsA and 20 μM of DIDS were used for further studies due to the fact that the 100–200 μM concentration range of DIDS led to significant reduction of bacterial number in culture (Data not shown). There was no inhibitory effect in range of 20–50 μM.

### Inhibition of VDAC-1 channel

Approximately, 1 × 10^5^ THP-1 macrophage-like cells were seeded in 24-well plates and pre-treated with either 5 μM CsA or 20 μM DIDS for 4 h. Cells were then infected with *M. avium* 104 for 2 h at MOI of 10:1, washed 3 times with HBSS and replenished with new RPMI medium supplemented with 10% FBS but without CsA or DIDS. Macrophages were lysed with 0.1% Triton X-100 at 4 h, day1, 2 and 3 post-infection, plated and CFUs were determined.

### Inactivation of VDAC-1 by siRNA

THP-1 cells were seeded at 60% confluence in 6-well plates and, 24 hours prior infection, transfected with control (scrabbled sequences) as well as experimental (VDAC-1) siRNAs purchased from Santa Cruz Biotechnology. Briefly, siRNAs were diluted in DMEM without serum at a final concentration of 25 nM and 3μl of Continuum^TM^ transfection reagent (Gemini) was added into diluted siRNA. The transfection mixture was added drop-wise to monolayers and then incubated at 37 °C in presence of 0.5% CO_2_ for 24 h. Next day, cells were infected with *M. avium* for 4 h, 1d, 2d, and 3d and CFUs were recorded on Middlebrook 7H10 agar plates. The VDAC-1 and β-actin protein levels from control and experimental wells were analyzed by semi-quantitative Western blotting on the Odyssey Imager (Li-Cor).

### Western Blot

Samples were mixed with an equal volume of 2X Laemmli sample buffer (Bio-Rad), resolved onto SDS-PAGE gel (Bio-Rad) and transferred to a nitrocellulose membrane (Bio-Rad). Membrane was blocked with 3% bovine serum albumin (BSA) in phosphate buffered saline (PBS) overnight. After, the membrane was incubated with primary antibody at a dilution of 1:250 for 2 h. Membrane was washed three times with PBS and then probed with corresponding IRDye secondary antibody (Li-Cor Biosciences, Inc) at a dilution of 1:5000 for 1 h. Proteins were visualized using Odyssey Imager (Li-Cor).

### The Yeast Two-Hybrid interaction

The VDAC-1 gene was fused in frame with the GAL4 DNA binding domain by inserting the PCR-generated fragment into the *Eco*RI and *Bam*HI sites of pGBKT7 (Clontech). The resultant bait vector pGBKT7:VDAC-1 was transformed into *Saccharomyces cerevisiae* strain Y2HGold using Yeastmaker Yeast Transformation System 2, according to the manufacturer’s instructions (Clontech). The following *M. avium* genes: MAV_1177, MAV_2921, MAV_2941 and CipA (MAV_4671) encoding secreted proteins identified in previous studies^3, 5, 34^ were fused with the GAL4 activation domain of pGADT7 and transformed into the yeast strain Y187 (Clontech). In addition, protein interaction experiments were extended for mmpL4 lipoproteins MAV_0084 and MAV_4996 as well as ATP synthase subunits of alpha (MAV_1525) and beta (MAV_1527) following the MS analysis of bacterial proteins from Table 2 and pull-down assay (Table 3). Plasmids pGBKT7–53, pGBKT7-lam, and pGADT7-T were used as positive and negative controls (Clontech). One ml of bait strain was combined with the one ml of prey strain and was grown in 2xYPDA liquid medium containing 50 μg/ml kanamycin at 30 °C for 24 h. The yeast zygotes that grew of blue color on Quadruple Dropout agar plates in absence of tryptophan (Trp), leucine (Leu), histidine (His), adenine, (Ade) (SD–Ade/–His/–Leu/–Trp) that contained 20 mg/ml X-a-Galactosidase and 125 ng/ml Aureobasidin were identified as positive clone.

### Immunoprecipitation of VDAC-1 and pull-down assay

THP-1 cells were lysed and proteins were precleared as previously described^44^. Ten microgram of anti-VDAC-1 mouse monoclonal antibody (Santa Cruz Biotechnology) conjugated to agarose beads was added to approximately 500μg of total cellular protein and incubated at 4 °C for 4 h. The sample was centrifuged at 2,500 rpm for 1 min and washed 3 times with PBS. The captured VDAC-1 was then added to *M. avium* total cell proteins and incubated at 4 °C for overnight. Twenty-four hours later, sample was washed three times with PBS and processed for tryptic Digestion (Thermo Fisher Scientific). Protein sequencing was performed at the Oregon Health and Science University proteomics facility by electrospray ionization mass spectrometry (ESI-MS/MS).

### Statistical analysis

All data are presented as ± SD and comparison of variables is performed using the unpaired Student’s *t* test. Statistical significance was set at *P* < 0.05.
